# Integrative Control of Energy Balance and Reproduction in Females

**DOI:** 10.5402/2012/121389

**Published:** 2012-09-26

**Authors:** R. M. Garcia-Garcia

**Affiliations:** Physiology Department (Animal Physiology), Complutense University, Avenida Puerta de Hierro S/N, 28040 Madrid, Spain

## Abstract

There is a strong association between nutrition and reproduction. Chronic dietary energy deficits as well as energy surpluses can impair reproductive capacity. Metabolic status impacts reproductive function at systemic level, modulating the hypothalamic GnRH neuronal network and/or the pituitary gonadotropin secretion through several hormones and neuropeptides, and at the ovarian level, acting through the regulation of follicle growth and steroidogenesis by means of the growth hormone-IGF-insulin system and local ovarian mediators. In the past years, several hormones and neuropeptides have been emerging as important mediators between energy balance and reproduction. The present review goes over the main sites implicated in the control of energy balance linked to reproductive success and summarizes the most important metabolic and neuroendocrine signals that participate in reproductive events with special emphasis on the role of recently discovered neuroendocrine peptides. Also, a little overview about the effects of maternal nutrition, affecting offspring reproduction, has been presented.

## 1. Introduction

The relationship between energy balance and reproduction is well known. Nutrition has a significant impact on numerous reproductive functions including hormone production, folliculogenesis, fertilization, and early embryonic development [1–3]. This intimate association is because reproductive processes are energetically expensive, and the brain must temper the fertility of individuals to match nutritional availability [4, 5]. Reproduction function in mammals can be inhibited when food availability is low or when increased energy demands are not met by compensatory food intake such as in short-term and chronic withdrawal of nutrients [6–8]. This very close alignment with the food supply is more important in females, where pregnancy and lactation are linked to considerable energetic expenses, needed for the nurture of embryos and newborns [9]. In fact, her reproductive outcome can be seriously altered and even life threatening to both the mother and offspring when nutritional imbalance occurs [4].

The link between nutrition and reproduction is mainly through the energy balance [1, 4], apart from the effect of specific nutrients that act independently of such energy balance [10]. Energy balance is usually measured as energy input, considered as feed intake, minus energy output that includes milk, maintenance, activity, growth, and pregnancy expenses [9, 11]. In order to keep constant body energy stores, in mammals, a series of homeostatic events leading to maintenance of energy balance are activate when a state of energy scarcity or abundance occurs. 

High yielding animal producers like high-producing dairy cows or primiparous rabbits are often in a serious negative energy balance (NEB) in some physiological phases, that is, lactation, since the net nutrient requirements are more elevated than the net nutrient intake in that stages (cows: [12, 13]; rabbits: [14]). NEB provokes low reproductive performance. Most of the studies about the influence of NEB have been performed on bovine species. It is well known that the metabolic condition of cows in NEB shifts to catabolic metabolism, which in turn causes increased plasma growth hormone and nonesterified fatty acid concentrations and decreased plasma insulin-like growth factor-I, insulin, and glucose concentrations [15–17] as well as leptin serum concentrations [18]. Also, compromised metabolic status decreases preovulatory follicle function [19], by means of intrafollicular changes such as production of low estradiol concentrations and IGFBP [20, 21]. This can be probably because cholesterol transport into the mitochondria to initiate steroidogenesis is affected [21]. In any case, changes in the growth pattern of the ovarian follicle during a period of NEB can indirectly affect oocyte quality that is ultimately responsible for the subfertility in dairy cows [22]. In other species with high requirements in early postpartum period like primiparous rabbit does, they show a profound NEB which negatively impacts on ovarian follicular and oocyte quality, giving rise to a poor reproductive performance too [14, 23].

In several species, long-term (chronic) and short-term (acute) undernutrition has been observed to suppress female reproduction through the suppression of Gonadotrophin-Releasing Hormone (GnRH) secretion, the delay of onset of puberty, the interference with normal estrous cycles, and the alteration of endocrine function (gilt: [24]; heifer: [25, 26]; ewe: [27]; rabbit: [28]). In this sense, inhibition or delay of preovulatory surge release of LH, decrease of oestradiol-17*β* discharge [1, 29] and increase of serum progesterone concentrations [30] have been described. Also, undernutrition affects ovarian follicle development [31], ovulation [32], blastocyst formation [33], and fertility rates [5, 34]. 

In contrast, when the nutritional requirements are less than the food supply, the animal will store the excess of nutrients (glycogen, triglycerides), being in a positive energy balance status. However, reproductive abnormalities are also common in obese individuals although mechanism behind this effect is unclear. In the genesis of obesity-induced infertility, pituitary insulin signaling seems implicated [35, 36]. Furthermore, recent advances in several species have been demonstrated that obesity negatively impacts the developmental competence of oocytes [37, 38]. Nevertheless, short-term energy supply termed as “flushing” around conception can improve ovulation rate and litter size [39] mainly in small ruminants. Flushing enhances follicle recruitment and follicle growth (for review see [4]). However, a high level of feeding is only beneficial to oocytes from animals of low body condition, because it is detrimental to oocytes in animals of moderate-to-high body condition [40]. 

The mechanisms mediating the influence of metabolism and nutrition on fertility are currently unclear, but there is a strong association between metabolic disorders and infertility [41]. It is difficult to determine the specific functions and mechanisms by which nutrition influences reproductive function. Circulating factors and hypothalamic circuits coordinate these responses in a complex manner. It is well known that the effects of nutrition are either mediated directly through effects on hypothalamic GnRH or pituitary gonadotropin secretion or indirectly through the growth hormone-IGF-insulin system or local ovarian mediators [42]. In the past years, several hormones and neuropeptides have demonstrated their important role as local mediators of brain to arbitrate the link between energy balance and reproduction. Understanding the integrative control of energy balance and reproduction can lead to reproductive success that will have benefits to improve clinical outcomes and farm animal production systems. The present review goes over the main sites implicated in the control of energy balance linked to reproductive success and summarizes the most important metabolic signals that participate in reproductive events with special emphasis on the role of recent discovered neuroendocrine peptides. A little overview about the intergenerational effects of nutrition affecting offspring has been presented.

## 2. Sites Implicated in the Control of Energy Balance Linked to Reproduction

**Figure 1:**
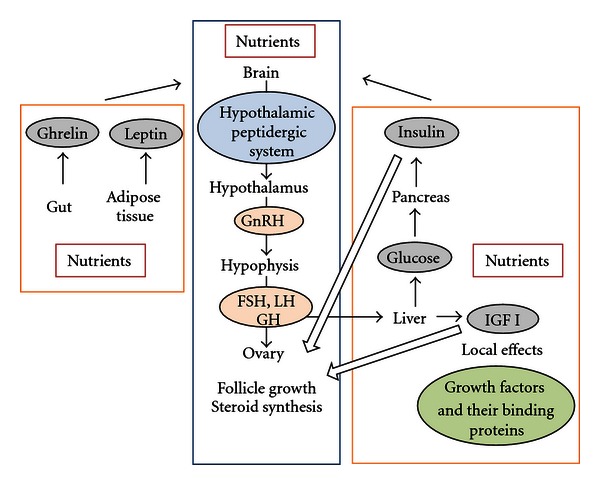
Schematic representation of mechanisms by which nutrition influences reproductive function.

Circulating levels of nutrients and metabolites, frequently, are not directly related to any of the factors that determine their concentrations but are related to a physiologically relevant combination of factors including nutrient reserves, nutrient intakes and nutrient demands for maintenance, growth, or production [43]. An enormous selection of chemical messengers and metabolic processes is involved in maintenance of energy balance and reproductive processes [8]. Most of these factors influence the hypothalamic-pituitary-gonadal (HPG) axis (for review see [8]). Potential sites of action of nutrition on ovarian function include systemic effects at the hypothalamic level via GnRH synthesis and release; the anterior pituitary through control of synthesis and release of FSH, LH, and growth hormone (GH); at the ovarian level through regulation of follicle growth [44] and steroid synthesis [45]. There are also possible local sites of action via effects on the cascade of growth factors and their binding proteins within the ovary [42, 45]. See Figure 1.

### 2.1. Hypothalamic-Pituitary Axis

A multilevel regulatory activity of autonomic centers and neuronal pathways is a noteworthy characteristic of many hypothalamic structures. The same groups of neurons are simultaneously involved in the performance of many regulatory functions. They are responsible for maintenance of energetic and osmotic homeostasis and also involved in the central regulation of reproductive processes, including sexual maturation and mating behavior [46].

The brain uses adipose- and gut-derived hormones, such as leptin, insulin, and ghrelin, to modulate the activity of the GnRH neuronal network that drives reproduction [5]. Recent studies had revealed that the major mechanism whereby the metabolic status impacts reproductive function involves modulation of the GnRH neuronal network at the hypothalamus [5, 8, 47], mainly by the multiple hypothalamic peptidergic systems responsible for the homeostatic control of energy balance [48] (this topic is reviewed in the following). Besides, metabolic challenges modify the GnRH, LH, and FSH surge, independently of their effects on pulsatile LH secretion [49].

### 2.2. Ovary

The ovary can respond directly to metabolic inputs independently of gonadotropin drive [50]. One of the more important events modulated by energy balance is folliculogenesis. The ovarian follicle is an integral part of the reproductive process. It has a major role in controlling the oestrous cycle, determining oestrous behaviour, ensuring oocyte competency and subsequent embryo survival rate, and determining both postovulation corpus luteum function and progesterone synthesis [42]. On the other side, gonadal function is regulated by the precise and coordinated secretion of the pituitary gonadotropins, luteinizing hormone (LH) and follicle-stimulating hormone (FSH) which is also modified by nutrition. In summary, folliculogenesis is a very complex but finely tuned process, in which endocrine and paracrine signals play an important role (for review, see [51]).

Folliculogenesis is stimulated by energy; particularly glucose is the main component of diet implied, although energy derived from fatty acid oxidation also appears to be important. On the other hand, proteins, vitamins, and other micronutrients probably exert permissive rather than regulatory functions on folliculogenesis [52]. The metabolic modulatory systems in follicular response are insulin-glucose, leptin and insulin-like growth factors (IGF) I and II and their binding proteins [53–55], which interact among them in a complex manner [4]. They are likely to be important mediators of the effects of dietary intake and/or energy balance [42]. 

Direct nutritional influence on ovary function depends on IGF-I from liver and on IGFBP concentrations, besides the reduction of follicular responsiveness of LH provoked by insulin suppressing follicular estradiol production below the threshold necessary to induce preovulatory-GnRH surge [8, 42]. For example, in gilts, feeding increases responsiveness of the ovary to LH through increasing insulin and IGF-I concentrations [56]. In ewes, nutrition changes can modulate the ability of gonadotrophin-dependent follicles to use the small amounts of FSH at the final stages of follicle growth, which are the most sensitive to low FSH levels [57]. 

## 3. Metabolic Signals That Control Energy Balance Linked to Reproduction

In general, plasma concentrations of some hormones such as insulin [58], IGF-I [59], and leptin [60, 61] augment when nutritional status improved by higher food intake or increased body fat depots. Conversely, they decrease with reduced food intake or increased tissue mobilization in pregnancy or lactation phases. 

Available metabolic fuels modulate hormone input to GnRH neurones, leading in turn to altered GnRH release and appropriate drive to the gonads [5]. In next section a review of the main hormones and neuropeptides implicated in the control of nutrition and reproduction is showed.

### 3.1. Somatotropic Axis Hormones, Insulin, and Glucose

Somatotropic axis hormones consist of growth hormone (GH), the insulin-like growth factors I and II (IGF-I and IGF-II), GH binding protein (GHBP), IGF binding proteins (IGFBPs) 1 to 6, and the cell-surface receptors for GH. GH as well as systemic and locally produced IGF can exert stimulatory, synergistic, or permissive effects at each level of the HPG axis, in the reproductive tract, external genitalia, and mammary gland [62]. This group of hormones has major effects on growth, lactation, and reproduction [63] and has a clear link with the metabolic status of the animals. Indeed, actions of GH and adequate levels of IGF-I in peripheral circulation are required to reach puberty and full reproductive potential [64].

GH seems to have a facilitatory rather than an obligatory role in reproduction [65]. It has direct effects on the follicle as well as indirect effects mediated by shifts in nutrient metabolism, insulin sensitivity, IGF-I, and IGFBP [64]. 

There are multiple levels of HPG axis at which IGF-I can act to coordinate reproduction with growth. In this sense, IGF-I acts as a direct regulator of GnRH neuron, usually excitatory [66]. In addition, IGF-I can also regulate the HPG axis via actions at the pituitary [67] and gonadal levels [68]. For that reason, IGF-I is a potential link between the reproductive and somatotropic neuroendocrine systems [66]. Additionally, local production of IGF-I independently of GH plays an important role in the intricate paracrine control of function of different types of somatic cells in the ovary. Insulin growth factor I and the IGF binding proteins (IGFBPs) participate by means of various mechanisms in different stages of follicular development, follicular steroidogenesis [51, 69], and oocyte maturation [70] as in the control of ovulation [71]. Therefore, insulin and IGF-I may be mediators between body condition and ovarian follicle development, ovulation and embryo development [50]. In fact, nutritionally induced changes in the ovarian IGF system play a key role in regulating oocyte quality [72].

Insulin is a modulator of the metabolic stimulus, rather than a mediator between the level of internal energy and the central effectors [8]. The common signaling pathway IGF-I receptor and insulin receptor (IR) signal through the insulin receptor substrate (IRS) proteins [73, 74] plays a role in regulating fertility under normal chow-fed conditions. Insulin has a direct effect at the ovarian level [4, 75]. Dietary restriction and NEB reduce circulating concentrations of insulin [76] and therefore could reduce androgen and estradiol production [77] which compromise the ability of follicles to acquire LH receptors [42]. Concentrations of insulin and IGF-I increase after a short-term supplementation, and they increase responsiveness to gonadotrophins, stimulate follicular growth, and suppress apoptosis in follicles [12, 53, 78]. It is unknown which insulin profiles are optimal for good follicle quality and uniformity, and how these insulin profiles can be achieved [79]. However, insulin secretion pattern can also be modulated by diet composition and feeding frequency. In this sense, the modulation of plasma insulin levels by dietary carbohydrates seems possible in sows, but IGF-I levels are less easily modified [80]. However, supplementation of fatty acids in cow did not improved plasma insulin concentrations [81].

Availability of locally produced IGF II in follicles is controlled by locally produced IGF-binding proteins (IGFBPs) [82]. IGF-II can modify the function of follicular cells by changes in diet which altered IGFBP-2 and IGFBP-4 [2]. Low blood concentrations of IGFBP occurred by undernutrition [83] or severe NEB [84], limiting the availability of IGF to target cells in the follicle and regulating their ability to stimulate cell proliferation and steroidogenesis [85].

Glucose is a very important mediator of nutritional effects on reproduction. Blood concentrations are inversely correlated to energy intake [86]. Glucose is transported by the family of facilitative glucose transporters (GLUTs) which get involved in hypothalamic regulation [87] and also plays a major role in providing metabolic substrates to oocyte and embryo [88, 89]. Glucose availability influences LH secretion through GnRH system [90]. Actually, numerous studies support the idea that glucose in particular mediates the effects of fasting to suppress GnRH-stimulated LH release. GnRH neurons might directly sense changes in glucose availability by a mechanism involving AMP-activated protein kinase [91]. On the other hand, glucose is the most important energy substrate for mammalian oocytes and blastocysts, so glucose deficiency can compromise the ability of the oocyte to reach the second metaphase, to extrude the first polar body [92] and to achieve the blastocyst stage. The changes in the role of glucose during preimplantation embryo development indicate that a specific interplay exists between glucose metabolism and the glucose transporters during different stages of preimplantation embryo development [93]. Besides, alterations in glucose transport and metabolism at the earliest stages of development can impact fetal development [88].

### 3.2. Adipokine Family: Leptin, Resistin, and Adiponectin

Adipokine family includes leptin, resistin, and adiponectin. Normal levels of adipokines are fundamental to maintain integrity of HPG axis, regular ovulatory processes, successful embryo implantation, and physiologic pregnancy [94]. 

Leptin is a 16 kD protein consisting of 146 amino acids which is synthesized primarily by adipose tissue. This protein was first identified as the gene product found to be deficient in the obese ob/ob mouse [95]. It is considered a potent satiety factor [96], and their concentrations in plasma reflect the amount of body fat [97]. Leptin modulates a diverse range of biological functions, including energy homeostasis and reproduction [98, 99]. In fact, the impact of leptin on feed intake, neuroendocrine-axis, and immunological processes has been demonstrated [100]. For that reason, leptin has been considered as the key link between nutrition and reproduction, like the appropriate signal to inform the reproductive system about the metabolic status [99]. 

Leptin is a permissive signal for puberty onset [101, 102], since a threshold of leptin is necessary for normal puberty to occur [103, 104]. The hypothalamus is an important site of leptin's action. Leptin is a potent stimulator of central GnRH and gonadotropin secretion [105, 106]. In addition, leptin has a direct effect on ovary being a potent inhibitor of ovarian steroidogenesis [107, 108]. In the ovary, leptin antagonizes the stimulatory effect of insulin on theca cell steroidogenesis, ultimately leading to a decrease in oestradiol secretion [109]. It also affects oocyte maturation [110], follicle rupture, corpus luteum formation [111], embryo implantation, and pregnancy [112]. Last study of Zhang et al. [113] suggests that role of leptin can be mediated by divergent modulation by gonadotropins even of a direct ovary effect.

Leptin concentrations are sensitive to short-term alterations in food intake and energy balance [114, 115]. Leptin presents the ability to increase fuel oxidation [8], influences whole-body glucose homeostasis and the action of insulin. Insulin sensitivity is impaired by leptin [116], and leptin production indirectly increases by insulin since insulin stimulates the secretion of leptin by adipocytes and by promoting lipogenesis [117]. 

The important role of leptin as link between nutrition and reproduction could be evidenced since although GnRH neurons do not express leptin receptors under physiological conditions, leptin influences GnRH neuron activity via regulation of immediate downstream mediators including the neuropeptides neuropeptide Y and the melanocortin agonist and antagonist (alpha-MSH and agouti-related peptide) [118, 119]. Besides, leptin promotes GnRH function via an indirect action on kisspeptin neurons [120, 121]. 

The other two adipokines have been less studied, and the central roles of adiponectin and resistin are less clear. Resistin impairs insulin sensitivity as leptin, whereas adiponectin enhances it. Adiponectin significantly reduced GnRH secretion [122] and inhibits GH and LH release [123]. In the ovary, adiponectin stimulates steroidogenesis by granulosa cells [116]. Resistin is expressed in rat and bovine ovaries and can modulate granulose cells functions in basal state or in response to IGF-I *in vitro* [124]. Resistin preferentially inhibits steroidogenesis of undifferentiated (small follicle) granulosa cells and inhibits proliferation of differentiated (large follicle) granulosa cells, indicating that the ovarian response to resistin is altered during follicular development [125].

### 3.3. Ghrelin

The peptide ghrelin was discovered by Kojima et al. [126] and identified an acylated 28 residue peptide released from the gut as the endogenous bioactive ligand for the growth hormone secretagogue receptor (GHS-R) type 1a. Ghrelin is involved in a wide spectrum of biological functions, including GH secretion and energy balance regulation, and exhibits diverse effects, including ones on glucose metabolism and on secretion and motility of the gastrointestinal tract [127]. The role of ghrelin in metabolism is the regulation of energy homeostasis, promoting food intake and weight gain [128]. The net orexigenic effect of ghrelin results is functionally opposite to that produced by leptin [129], and many data support the notion that both hormones act in a complementary fashion in providing the central nervous system information about the energy balance for the maintenance of homeostasis [130–132]. 

Serum ghrelin levels are influenced by both short- and long-term changes in energy homeostasis (i.e., with glucose, insulin, and somatostatin levels) [133]. Ghrelin is considered as a signal of starvation or energy insufficiency with a negative correlation observed between body mass index (BMI) and ghrelin levels. In this sense, normal timing of puberty can be delayed if ghrelin levels are persistently elevated as putative signal for energy insufficiency [118]. In the control of the reproductive function, ghrelin shows a complex mode of action upon the gonadotropic axis, with predominant inhibitory effects at central (hypothalamic) levels and upon GnRH-induced gonadotropin secretion, but direct stimulatory actions on basal LH and FSH secretion [134]. Gonadal physiology is also regulated by grhelin [118, 127, 135] and even in preimplantation period [133] although the mechanism remains unclear to date.

### 3.4. Neuropeptides and Modulators

Neuropeptides including neuropeptide Y (NPY), products of the proopiomelanocortin (the proopiomelanocortin-(POMC-) derived peptide and alpha-melanocyte-stimulating hormone (alpha-MSH)), galanin-like peptide (GALP), and kisspeptins are thought to be implicated in the control of metabolism and reproduction. The involvement is supposed because neurons that express these neuropeptides all reside in the hypothalamic arcuate nucleus, a critical site for the regulation of both metabolism and reproduction. In addition, these neuropeptides are all targets for regulation by metabolic hormones, such as leptin and insulin. And finally, these neuropeptides have either direct or indirect effects on feeding and metabolism, as well as on the secretion of GnRH and LH [136]. The discovery of kisspeptins and gonadotropin-inhibitory hormone (GnIH) sheds a new light on mechanisms by which reproductive activity is regulated. Other molecules such as the GnRH II, orexins, and nesfatin-1 have revealed also a role in the control of metabolism and reproduction.

#### 3.4.1. Neuropeptide Y (NPY)

Neuropeptide Y, the most potent orexigenic peptide known [99], is a 36-amino acid peptide neurotransmitter. It has a dual function in relation to reproduction and appetite [137]. It stimulates food intake and negatively regulates reproduction [138, 139] because it inhibits LH secretion [140, 141]. NPY seems to be implicated in the generation of the preovulatory surge of LH [142].

#### 3.4.2. Melanocortin System

The melanocortin system, involving melanocyte stimulating hormone, adrenocorticotropic hormone, agouti-related peptide and the central melanocortin 3 and 4 receptors, plays a major role in the hypothalamic regulation of energy balance [143]. Conversely to NPY, melanocortin signaling controls ingestive behavior, energy balance, and substrate utilization [99] by means of reducing food intake and stimulating reproduction [144, 145]. Melanocortin has recently demonstrated to be an important component in the leptin-mediated regulation of GnRH neuron activity, initiation of puberty and fertility [119, 146]. 

#### 3.4.3. Galanin-Like Peptide (GALP)

Galanin-like peptide (GALP) is a 60-amino acid neuropeptide which belongs to the G protein-coupled receptors (GPCRs) family. GALP is mainly produced in neurons in the hypothalamic arcuate nucleus. The effects of GALP on food intake and body weight are complex. In rats, the central effect of GALP is to first stimulate and then reduce food intake, whereas in mice, GALP has an anorectic function. Furthermore, GALP shows direct stimulatory action on gonadotropin secretion [147, 148], regulates plasma LH levels through activation of GnRH producing neurons, suggesting that it is also involved in the reproductive system [148, 149]. The presence of galanin within kisspeptin axons innervating GnRH neurones and the oestrogen-dependent regulation of that presence add a new dimension to the roles played by galanin in the central regulation of reproduction [150]. 

#### 3.4.4. Kisspeptin

The Kiss1 gene encodes a family of peptides called kisspeptins, which bind to the G protein-coupled receptor GPR54. Humans and mice with loss-of-function mutations of the genes encoding kisspeptins (Kiss1) or kisspeptin receptor (Kiss1r) are infertile due to hypogonadotropic hypogonadism [151, 152]. The results from a wide variety of studies indicate that kisspeptin stimulates gonadotropin secretion via a hypothalamic pathway that activates GnRH neurons. Kisspeptins have emerged as important gatekeepers of key aspects of reproductive maturation and function, from sexual differentiation of the brain and puberty onset to adult regulation of gonadotropin secretion and the metabolic control of fertility (for review see [153]).

Hypothalamic Kiss1 neurons are highly sensitive to body energy status and metabolic cues, as evidenced by suppressed Kiss1/kisspeptin expression in conditions of negative energy balance, which are also linked to inhibition of the reproductive axis [154]. Kisspeptin neurons are downstream mediators of leptin's positive effect on the secretion of gonadotropins [154], and it is affected by leptin status [155]. Besides, network between Kisspeptin cells communicating with NPY and POMC cells seems to coordinate brain control of reproduction and metabolic homeostatic systems [155, 156]. Agonists and antagonists of kisspeptin have emerged as valuable new tools for manipulating the HPG axis and are promising drugs for future treatment [157].

#### 3.4.5. Gonadotropin Inhibitory Hormone (GnIH)

The gonadotropin inhibitory hormone (GnIH) acts via the novel G protein-coupled receptor 147 (GPR147) to inhibit gonadotropin release and synthesis. It has also a dual role with a function in the regulation of reproduction and food intake [158]. It stimulates food intake in rats [159], and recent data indicate a direct action of GnIH on the pituitary gonadotrope to reduce both synthesis and secretion of LH (160), so it could be considered as a blocker of reproductive function in mammals [160]. Recent evidence further indicates that GnIH operates at the level of the gonads as an autocrine/paracrine regulator of steroidogenesis and gametogenesis (for review see [161]).

#### 3.4.6. GnRH II

One form of gonadotropin releasing hormone (GnRH) now called GnRH II acts as a permissive regulator of female reproductive behaviour based on energy status, as well as a modifier of short-term food intake [162]. GnRH II plays a critical role by orchestrating the coordination of reproduction with the availability of nutritional support [163]. 

#### 3.4.7. Orexins

Orexins A and B are neuropeptides which are synthesized mainly in the lateral hypothalamus and are associated with a variety of physiological functions such as energy homeostasis and reproduction. The orexins activate two G-protein-coupled receptors termed orexin receptor 1 (OX1R) and orexin receptor 2 (OX2R). They are implicated in the regulation of GnRH cells [164] as a mechanism whereby leptin can influence reproductive neuroendocrine function. Also, orexin A is implicated in pulsatile LH secretion [165] which is potentiated by estrogen [166].

#### 3.4.8. Nesfatin-1

Nesfatin-1 (NEFA/NUCB2-encoded satiety and fat-influencing protein) is a recently discovered and still relatively unknown hypothalamic peptide which can be considered as one of the regulatory factors of the hypothalamic-pituitary-axis. Nesfatin-1 is a potent anorexigenic factor inducing satiety and strongly inhibiting food and water intake [167, 168]. It is implicated in the gonadotropin secretion during puberty [169], and these processes can be greatly disturbed by negative energy balance, caused by a short-term starvation or nutritional deficiency [170]. Further studies are required to involve Nesfatin-1 in regulation of gonadotropin secretion in adulthood [46].

## 4. Effect of Maternal Nutrition on Fetal and Neonatal Reproductive Development and Function 

Maternal nutrition, mainly in periconceptional period, can have long-term consequences on health and well-being of the offspring. That has been termed developmental programming. In livestock, developmental programming affects production traits, including growth, body composition [171], and reproduction [172, 173]. Latest studies are indicating the very important role of maternal nutrition on offspring development given that reproductive performance is clearly influenced by prenatal factors. Mechanisms by which environmental factors affect the reproductive organs of developing offspring are not well known to date neither the future consequences of maternal nutrition. 

Maternal nutrition can influence development of the fetal reproductive system at all stages of development, during the processes of differentiation and development [174–176] and between birth and puberty [177, 178]; effects are exerted before neuroendocrine organs (like the hypothalamus and pituitary gland), and reproductive organs have been differentiated [179]. It involves many different physiological systems. Therefore, a wide range of mechanisms are involved (for review see [43]). For example, a recent study has elucidated that the sensitivity of Kiss1r mRNA, which is expressed in GnRH neuron, to nutritional status has been already established during the early neonatal period [180]. 

Scarce literature about effects of maternal nutrition on reproductive outcome of offspring has been reported. Nevertheless, maternal undernutrition and overnutrition or supplementation seems to impact on components of the HPG system of offspring [43]. For example, maternal undernutrition during the first month of pregnancy resulted in increased pituitary sensitivity to GnRH and increased number of small follicles in the ovary, while during mid to late gestation resulted in a reduction of large corpora lutea in female sheep offspring [181]. On the other hand, transgenerational supplementation with fish oil significantly decreased ovulation rate and litter size in female mice [182].

Further studies are required to better understand the impact of maternal nutrition on offspring future reproductive success. 

## 5. Conclusion

The finding of the protein leptin opened up a new time in the understanding of the neuroendocrine control of energy homeostasis and its close relationship with the reproductive axis [118]. In recent years, thanks to a dynamic development of molecular biology, a number of new regulatory neuropeptides have been identified and described. Recent discoveries have elucidated the important role of periphery factors, such as leptin, ghrelin, and insulin. However, they are integrated with a complex network of neuropeptides, whose actions are located upstream of the GnRH cell population in brain. For example, compelling evidence indicates that kisspeptins and their receptor represent key elements in the neuroendocrine control of reproduction. Besides, GnIH has fundamentally changed our understanding of hypothalamic control of reproduction. In addition, some local factors related to metabolic status are extremely important in ovarian regulation. In conclusion, integrative control of energy balance and reproduction is carried out by multiple metabolic and neuroendocrine signals that control reproduction in an intricate manner, even affecting next generations.
